# A dual-purification system to isolate mitochondrial subpopulations

**DOI:** 10.1242/jcs.263693

**Published:** 2025-04-14

**Authors:** Corey N. Cunningham, Jonathan G. Van Vranken, Jakeline Larios, Katarina Heyden, Steven P. Gygi, Jared Rutter

**Affiliations:** ^1^ Department of Biochemistry, University of Utah School of Medicine, Salt Lake City, UT 84112, USA; ^2^ Department of Cell Biology, Harvard Medical School, Boston, MA 02115, USA; ^3^ Howard Hughes Medical Institute, Salt Lake City, UT 84112, USA

**Keywords:** Mitochondria, Biochemistry, Purification, Proximity labeling

## Abstract

Mitochondria perform diverse functions, including producing ATP through oxidative phosphorylation, synthesizing macromolecule precursors, maintaining redox balance among many others. Given this diversity of functions, we and others have hypothesized that cells maintain specialized subpopulations of mitochondria. To begin addressing this hypothesis, we developed a new dual-purification system to isolate subpopulations of mitochondria for chemical and biochemical analyses. We used APEX2 proximity labeling such that mitochondria were biotinylated based on proximity to another organelle. All mitochondria were isolated by an elutable MitoTag-based affinity precipitation system. Biotinylated mitochondria were then purified using immobilized avidin. We used this system to compare the proteomes of endosome- and lipid droplet-associated mitochondria in U-2 OS cells, which demonstrated that these subpopulations were indistinguishable from one another but were distinct from the global mitochondria proteome. Our results suggest that this purification system could aid in describing subpopulations that contribute to intracellular mitochondrial heterogeneity, and that this heterogeneity might be more substantial than previously imagined.

## Teaser

**Summary:** A dual-purification scheme is used to isolate subpopulations of mitochondria based on their juxtaposition to various membrane-bound organelles. Some of these subpopulations have mitochondrial proteomes that are significantly different from an average proteome.

## INTRODUCTION

Mitochondria, once thought to only be the ‘powerhouse of the cell’, perform a variety of metabolic and non-metabolic functions that are required for normal cell biology as well as adaptation to stress and signaling. For example, mitochondria maintain cellular redox potential through a variety of oxidation and reduction reactions, and synthesize macromolecular precursors, such as nucleotides and amino acids (Suomalainen and Nunnari, 2024; Shen et al., 2022). Failure of any of these key functions can result in metabolic diseases, such as cancer, heart disease, myopathy and diabetes (Suomalainen and Nunnari, 2024). Thus, a deeper understanding of the biology of mitochondria and how they perform this variety of functions remains of high value.

Recently, several studies have explored the concept that cells have distinct and specialized populations of mitochondria. Many of these studies, however, are limited to the intermyofibrillar and subsarcolemmal mitochondria of skeletal muscle. Morphologically, subsarcolemmal mitochondria are larger and less branched than intermyofibrillar mitochondria (Vincent et al., 2019). Proteomic analyses have found that subsarcolemmal mitochondria have decreased electron transport chain (ETC) complex I and III subunit abundance but are enriched in stress-related proteins, such as HSPA9, when compared to intermyofibrillar mitochondria (Ferreira et al., 2010). Additionally, subsarcolemmal mitochondria have a higher β-oxidation capacity in both normal and endurance trained mice (Koves et al., 2005), suggesting that these two mitochondrial subpopulations might prefer different fuel sources to drive ATP production. Another profound example of intracellular mitochondrial heterogeneity was demonstrated in brown adipose tissue, where mitochondria segregate the seemingly incompatible mitochondrial functions of heat production and ATP synthesis between their cytosolic and peri-lipid droplet populations (Benador et al., 2018). In this scenario, peri-lipid droplet mitochondria produce ATP for triglyceride synthesis and lipid droplet expansion while having lower β-oxidation capacity. The cytosolic mitochondria, on the other hand, exhibit higher β-oxidation and uncoupling to produce heat (Benador et al., 2018).

Our growing understanding of the spatial regulation of mitochondrial maintenance and quality control supports the concept of subcellular populations of specialized mitochondria. For example, mitochondrial fission events and mtDNA replication specifically occur at endoplasmic reticulum (ER)–mitochondria contact sites (Lewis et al., 2016). Precise imaging strategies have also provided evidence of mitochondrial heterogeneity (Collins et al., 2002). For example, use of the cationic carbocyanine dye JC-1 has suggested that mitochondria membrane potential might be highly variable within a given cell (Collins et al., 2002). Comparatively, tetramethylrhodamine-based fluorescence cell sorting has also been used to isolate mitochondrial subpopulations based on membrane potential for downstream analyses (MacDonald et al., 2019). Fluorescence imaging has also captured mitochondrial permeability transition pore opening in distinct mitochondria subpopulations upon treatment with *tert-*butyl hydroperoxide (Collins et al., 2002), thereby highlighting the distinct responses of specific subsets of mitochondria.

Given these examples, we hypothesized that intracellular mitochondrial heterogeneity enables specialized subsets of mitochondria to fulfill distinct functions. However, whether these specialized subpopulations are the result of differing microenvironments or are the result of differential protein localization is unknown. With regards to the latter, a recent study has discovered that a subset of mitochondria contains pyrroline-5-carboxylate synthase (P5CS; also known as ALDH18A1) filaments (Ryu et al., 2024). Although these mitochondria have higher membrane potential when compared to their counterparts, these P5CS-containing mitochondria lack ATP synthase components, ultimately driving reductive synthesis pathways at the expense of OXPHOS (Ryu et al., 2024). Still, a comprehensive investigation into differential mitochondrial proteomics has not been described, as current techniques are limited to those that can be biochemically isolated, which is applicable to only a few subsets. Given this technological gap, we set out to design a dual-purification strategy to systemically isolate subpopulations of mitochondria for proteomic or lipidomic analyses. First, we generated cells wherein a specific mitochondrial population was biotinylated using APEX2 fusion proteins targeted to one specific organelle membrane (Lam et al., 2015). Each APEX2 system was methodically optimized in U-2 OS cells to avoid excessive biotinylation, which would limit specificity. Using a modified, elutable MitoTag affinity purification (AP) system, we rapidly purified all mitochondria to remove contaminants. Eluted mitochondria were then subjected to streptavidin affinity purification to capture the subpopulations that were biotinylated intracellularly. Using this system, we performed a pilot study to observe potential proteomic differences between mitochondrial subpopulations isolated using our endosomal–APEX2 and lipid droplet–APEX2 systems. Comparing both endosome- and lipid droplet-associated mitochondrial proteomes to the bulk mitochondrial population revealed that these subpopulations had proteomes that were distinct from the overall mitochondrial proteome isolated from their respective cell lines. Ultimately, we anticipate this dual-purification strategy could be used in future investigations that provide insight into mitochondrial specialization, such as the characterization of P5CS-containing subpopulation, aiding in the discovery of new aspects of mitochondrial biology.

## RESULTS

**Fig. 1.**
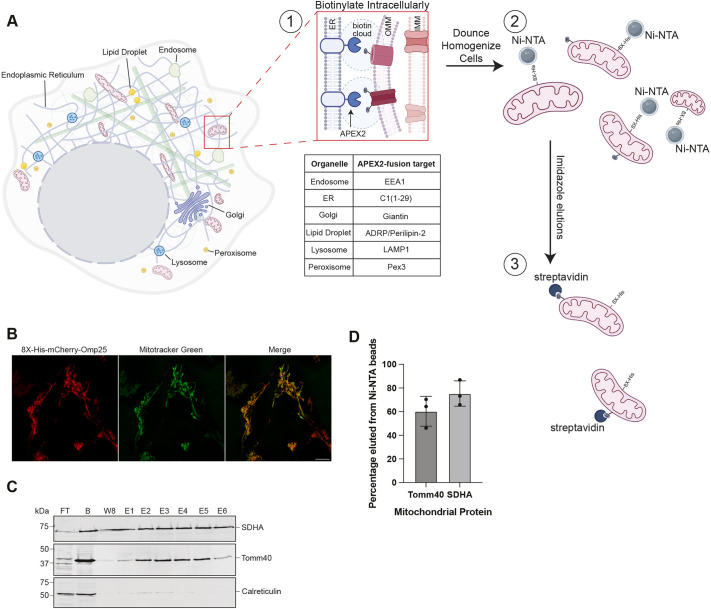
**A dual-purification system to isolate proximity labeled mitochondria.** (A) An illustration of a dual-purification system to isolate subpopulations of mitochondria. Step 1, APEX2 systems (listed in table) create a reactive biotin radical cloud (blue dotted circle) in the presence of hydrogen peroxide and biotin phenol. Mitochondrial proteins within the reactive biotin cloud will be biotinylated; step 2, 8× histidine (8X-His)-epitope-tagged mitochondria are purified by nickel-NTA beads and are eluted upon addition of imidazole; step 3, mitochondria biotinylated intracellularly in step 1 will be affinity purified by streptavidin-conjugated beads. Created in BioRender by Cunningham, C., 2025. https://BioRender.com/f34f381. This figure was sublicensed under CC-BY 4.0 terms. (B) Live-cell imaging of U-2 OS cells stably expressing 8×His–mCherry–Omp25 and MitoTracker Green (50 nM). Images representative of *n*=3 experimental repeats. Scale bar: 10 μm. (C) U-2 OS cells stably expressing 8×His–mCherry–Omp25 were affinity purified, subjected to eight washes, eluted, subjected to SDS-PAGE and immunoblotted with the indicated antibodies (*n*=3). FT, flow through; B, sample remaining on beads after six elutions, W8, eighth wash step, E1–E6, elutions 1 through 6. (D) Quantification of immunoblots in C with all elutions combined and divided by sum of elution total with amount of protein remaining on beads B. Data represent the mean±s.d. (*n*=3).

We reasoned that spatially segregated mitochondria might have distinct proteomes due in part to different microenvironmental factors. We hypothesized that mitochondrial subpopulations that are juxtaposed to other organelles might differentially conduct fuel usage and biosynthesis and respond to distinct signaling cues, which would be reflected in their protein composition. To test this, we designed a tool to systematically isolate mitochondria that are in proximity of different organelles. First, the ascorbate peroxidase, APEX2, was tagged with green fluorescent protein (GFP) and targeted by fusion with the cytosolic-facing portion of resident membrane proteins of various organelles, including endosomes (EEA1), ER (C1[1-29]; the first 29 amino acids of cytochrome P450), the Golgi (giantin; also known as GOLGB1), lipid droplets (ADRP; also known as perilipin-2), lysosomes (LAMP1) and peroxisomes (Pex3) (Fig. 1A; Fig. S1). We validated the organellar localization of each fusion protein (Fig. S1). Although APEX2 proximity labeling has an estimated biotin cloud of 50 nm in diameter (Fig. 1A), we expected that a single streptavidin affinity purification would not adequately enrich for mitochondria owing to the extensively labeled intrinsic proteins of each organelle. To address this issue and enrich only for mitochondria, we modified the MitoTag-IP system, which classically uses a 3× hemagglutinin (HA) epitope tag fused to GFP and the tail-anchored targeting sequence of Omp25 (Chen et al., 2016). To make it compatible with our approach, we exchanged the 3×HA epitope tag for an 8× histidine (His) tag, which enabled us to efficiently purify and elute mitochondria (Fig. 1B–D). This new elutable 8×His–mCherry-fused Omp25 colocalized with MitoTracker Green (Fig. 1B), with ∼60–75% of the total amount eluted after six elution rounds with imidazole, and specifically enriched for mitochondria (Fig. 1D). As an example of specificity, when the ER-resident protein calreticulin was bound to the nickel-NTA beads for His purification it did not efficiently elute (Fig. 1C). Taken together, these results encouraged us that our protocol of APEX2 proximity labeling (one organelle at a time), rapid His-tagged affinity purification for mitochondria enrichment and capture of the biotinylated mitochondria might be able to isolate select mitochondrial populations that resided adjacent to various organelles (Fig. 1A).

**Fig. 2.**
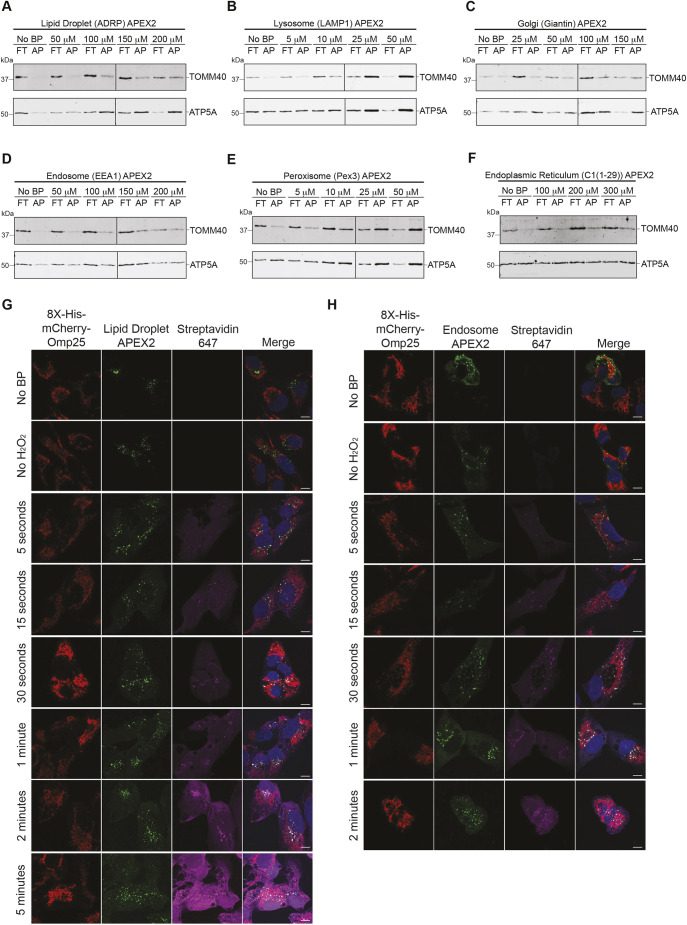
**Timing and specificity of APEX2 fusion proteins and optimization of dual-purification systems in U-2 OS cells.** (A) U-2 OS cells stably expressing 8×His–mCherry–Omp25 and the ADRP–APEX2 system were dual purified. After imidazole elutions, mitochondria were subjected to streptavidin affinity purification (AP). One-half of the total sample loaded onto SDS-PAGE was subjected to immunoblotting with the indicated antibodies. Flow-through (FT) indicates mitochondria not captured by streptavidin purification. Black line indicates splicing between two gels run and exposed at the same time. (B) As in A, but with the LAMP1-APEX2 system. Black line indicates splicing between two gels run and exposed at the same time. (C) As in A, but with the Giantin-APEX2 system. Black line indicates splicing between two gels run and exposed at the same time. (D) As in A, but with EEA1–APEX2 system. Black line indicates splicing between two gels run and exposed at the same time. (E) As in A, but with the Pex3-APEX2 system. Black line indicates splicing between two gels run and exposed at the same time. (F) As in A, but with the C1(1-29)–APEX2 system. All blots representative of *n*=3 experiments. (G) Immunofluorescence of fixed U-2 OS cells stably expressing 8×His–mCherry–Omp25 and the GFP–ADRP–APEX2–V5 (lipid droplet) system, labeled with 100 µM biotin phenol, for the indicated time points. Cells were stained with goat anti-streptavidin 647 (*n*=3). (H) As in G, but with the GFP–APEX2–V5–EEA1 (endosome) system. Images representative of *n*=5 experimental repeats. Scale bar: 10 μm.

Given that there are varying levels of expression of each proximity labeling system, we sought to optimize our protocol in the commonly used U-2 OS osteosarcoma cells (Fig. 2; Fig. S2). The goals of this optimization were to: (1) determine a biotin phenol concentration that labeled equivalently across APEX2 systems due to differing expression levels between the systems, and (2) determine the ideal timing of each APEX2 system. For each APEX2 system, biotin phenol concentrations from 5 to 300 µM were used (Fig. 2A–F). We aimed to label ∼15–20% of eluted mitochondria, using TOMM40 as a proxy for equal mitochondrial labeling (Fig. 2A–F, see ‘AP’ lanes). This strategy allowed us to find an optimized concentration of biotin phenol for each organelle-specific APEX2-labeling system. For instance, 5–10 µM of biotin phenol in the LAMP1–APEX2 lysosomal system labeled a mitochondrial fraction that was similar to 100 µM for both the EEA1–APEX2 endosomal and ADRP–APEX2 lipid droplet systems using TOMM40 capture on streptavidin beads as a readout (Fig. 2B, lanes 3 and 4 compared to Fig. 2A and D, lanes 5 and 6). Importantly, isolated mitochondria were mostly intact, with a minority of the mitochondria having a slightly disrupted outer membrane, as evidenced by slight HtrA2/Omi cleavage without hypotonic treatment (Fig. S2A).

As an important negative control, we found that each APEX2 system was ‘off’ in the absence of either biotin phenol, as measured by streptavidin 647 immunofluorescence (Fig. 2G,H, first row; Fig. S2B–E, first row), or hydrogen peroxide (Fig. 2G,H, second row; Fig. S2B–E, second row). Next, using the biotin phenol concentrations determined in Fig. 2A–F, we observed each that APEX2 fusion system was specific at timepoints from 15 s to 1 min. This specificity was demonstrated by the streptavidin 647 signal localizing proximal to the GFP fluorescence of each APEX2 system. Longer APEX2 labeling from 2 to 5 min resulted in substantially increased, but more diffuse, streptavidin signal (Fig. 2G,H; Fig. S2).

**Fig. 3.**
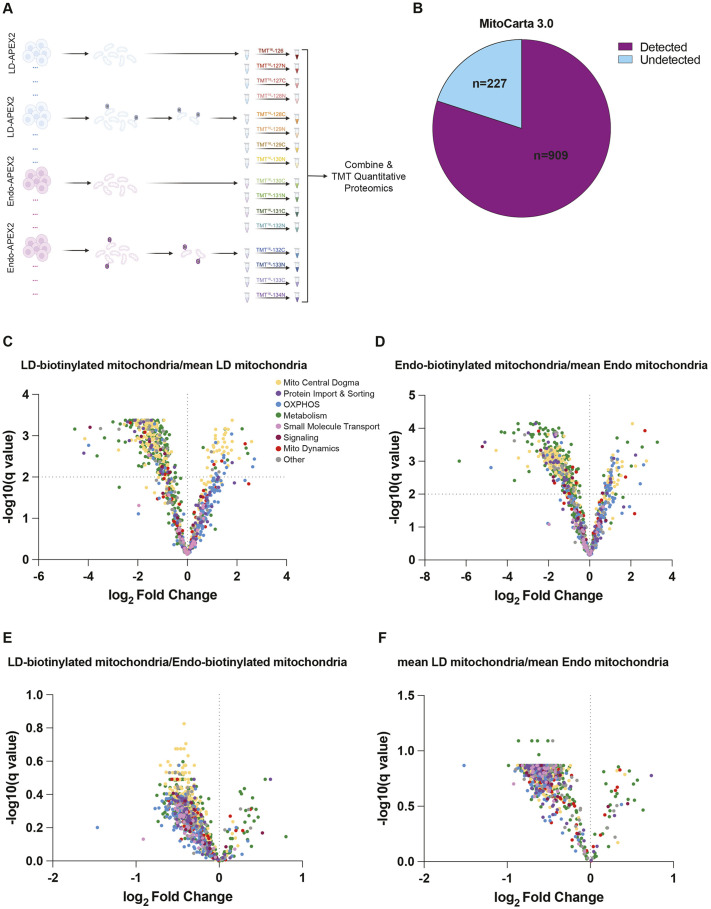
**Mitochondrial subpopulations proximal to endosomes and lipid droplets have similar proteomes, but both are significantly different from an average mitochondrial proteome.** (A) Illustration of TMT-quantitative proteomic strategy. Four biological samples of mean mitochondria from the ADRP–APEX2 system, four biological samples of ADRP–APEX2-biotinylated mitochondria, four biological samples of mean mitochondria from the EEA1–APEX2 system, and four biological samples of EEA1–APEX2-biotinylated mitochondria were isolated, labeled with the TMT-16 plex indicated labels, combined and analyzed via mass spectrometry. Created in BioRender by Cunningham, C., 2025. https://BioRender.com/cct94sg. This figure was sublicensed under CC-BY 4.0 terms. (B) Pie chart of TMT proteomics indicating the detection of 909 of the 1136 mitochondrial proteins observed on MitoCarta 3.0. (C) Volcano plot derived from TMT quantitative proteomics comparing mitochondrial proteins from mitochondria biotinylated by the lipid droplet (LD)–APEX2 system and dual purified compared to the mean mitochondrial proteome from the corresponding U-2 OS cell line. Each dot was colored according to their MitoPathway term from MitoCarta 3.0, with hierarchy of ‘Mitochondrial Central Dogma’ down to the lowest ‘Other’. (D) As in B, but mitochondrial biotinylated by the endosome–APEX2 system and dual purified compared to the mean mitochondrial proteome from the corresponding U-2 OS cell line. (E) Volcano plot comparing mitochondria biotinylated from the lipid droplet–APEX2 system and dual purified to mitochondria biotinylated from the endosome–APEX2 system and dual purified. (F) Volcano plot comparing the mean mitochondrial proteomes from the 8×His–mCherry–Omp25 U-2 OS cell lines expressing either lipid droplet–APEX2 or endosome–APEX2 systems. Data presented in volcano plots were FDR-corrected using the two-stage step-up method of Benjamini, Krieger and Yekutieli (Benjamini et al., 2006).

Because we observed a similar extent of labeling between the endosome–APEX2 (EEA1) and lipid droplet–APEX2 (ADRP) systems, we sought to assess whether mitochondrial subpopulations proximal to endosomes and lipid droplets had distinct proteomes from one another and from the general mitochondrial population in a cell. Hence, we analyzed the proteome of eluted labeled mitochondria from each of the two cell lines. As a control, mean mitochondrial populations from untreated cells of both systems were purified using our elutable MitoTag-AP system in the same APEX2 cellular background as the biotinylated populations. Once eluted, these mitochondria were subjected to downstream proteomics following a multiplexed TMT quantitative proteomics strategy (Fig. 3A).

Our proteomics analysis detected 909 of the 1136 mitochondrial proteins annotated in MitoCarta 3.0 (Fig. 3B) (Rath et al., 2021) and four of the top six gene ontology cellular component analyses (GOCC) were mitochondrial or relating to mitochondria (Fig. S3A). Interestingly, both endosome- and lipid droplet-biotinylated mitochondrial proteomes were distinct from their respective mean mitochondria proteomes, demonstrating that each had a distinct mitochondrial proteome (Fig. 3C,D). Of note, these two biotinylated mitochondrial proteomes were enriched for many OXPHOS components while being depleted for many metabolism proteins as shown by the MitoPathways clustered view (Fig. 3C,D) (Rath et al., 2021). Of note, many non-mitochondrial proteins were also significantly enriched or depleted in a specific population (Fig. S3B,C). In-depth analyses revealed that ATP synthase inhibitory factor subunit 1 (ATP5IF1) was almost completely excluded in our endosomal- and lipid droplet-biotinylated subpopulations. Interestingly, both peri-lipid droplet and peri-endosomal subpopulations had decreased abundances of proteins associated with stress responses, such as heat shock protein family E (HSPE1), superoxide dismutase (SOD2) and peptidylprolyl isomerase F (PPIF), compared to the mean mitochondrial proteome (Table S1).

Whereas the proteomes of both peri-lipid droplet and peri-endosomal mitochondrial subpopulations were quite different from the mean mitochondrial proteome, surprisingly there were no statistically significant differences with false discovery rate (FDR) correction when we compared these two mitochondrial proteomes to one another (Fig. 3E). Of note, there were several proteins that passed our *P*-value cutoff but were suppressed upon FDR correction, such as acetyl-CoA carboxylase (ACACA) and peroxiredoxin-6 (PRDX6) in the peri-lipid droplet subpopulation. Moreover, in the peri-endosomal subpopulation, we observed an enrichment in the mitochondrial rRNA methyltransferase 2 (MRM2), tRNA methyltransferase 5 (TRMT5) and several mitochondrial subunits, such as MRPL14, MRPL27 and MRPS28.

Importantly, no other global proteomic differences were observed when comparing these two populations (Fig. S3D). As predicted, there were no observable differences between the total mitochondrial proteomes from either cell line except for the increase in ADRP (Fig. 3F; Fig. S3E). It is interesting that EEA1, which was overexpressed, did not co-purify with mitochondria like ADRP.

## DISCUSSION

Intracellular mitochondrial heterogeneity has the potential to provide cells with the ability to segregate tasks, diversify fuel usage and adapt to stress. Recent evidence of such specialized subpopulations in brown adipose tissue and cardiac and skeletal myocytes prompted us to design a strategy to isolate subpopulations of mitochondria for proteomic or lipidomic analyses. In doing so, we sought to discover mitochondrial specialization on a broader scale and highlight nuances that might not be seen as morphological changes by microscopic techniques. Therefore, we created a dual-purification system, combining an elutable MitoTag affinity purification with spatially resolved APEX2 proximity labeling to isolate mitochondria subpopulations based on their subcellular localization. After optimizing this system (Fig. 2), we examined the proteomic differences between mitochondria in close proximity to endosomes and lipid droplets, while also comparing these proteomes to the average mitochondrial proteome. Our proteomic dataset had high coverage, capturing 909 of the 1136 (80%) mitochondrial proteins listed in MitoCarta 3.0.

When comparing mean mitochondrial proteomes from the lipid droplet–APEX2 stable U-2 OS cell line to the lipid droplet proximal mitochondria, the biotinylated mitochondria have increased abundances of OXPHOS complex V and ATP synthase components, like ATP5F1B, MT-ATP8 and ATP5MPL. This result is consistent with the observation that peri-lipid droplet mitochondria in brown adipose tissue have higher ATP synthesis rates than their cytosolic counterparts (Benador et al., 2018). It has been suggested that peri-lipid droplet mitochondria also impact their protein composition by being less mobile and limiting fusion events (Benador et al., 2018). It remains unclear why endosome proximal mitochondrial proteomes are so similar to lipid droplet proximal mitochondria. One hypothesis is that, like peri-lipid droplet mitochondria, mitochondria that are proximal to endosomes perform similar metabolic reactions or participate in less fusion events. We postulate that this latter hypothesis is not likely because endosomes are only thought to participate in the transferrin ‘kiss-and-run’ reaction with mitochondria (Das et al., 2016). It is possible that it is only under stress conditions, such as fatty acid overload, that proteomic differences between these two subpopulations could be observed.

Why are these two biotinylated mitochondria proteomes significantly different from the mean mitochondrial proteomes from their respective cells? One possibility is that one or more of the other mitochondrial subpopulations heavily influence the differences observed between the mean mitochondria proteome and the endosomal- and lipid droplet-mitochondria proteomes. A plausible organelle responsible for such a strong proteomic influence is the ER. The well-studied ER–mitochondrial contact sites are required for Ca^2+^ sequestration, mitochondrial dynamics, redox balance and reactive oxygen species production, and inflammation (Voss et al., 2012; Sassano et al., 2023; Rizzuto et al., 1993; Lee et al., 2016; Friedman et al., 2011; Booth et al., 2016; Zhou et al., 2011). Each of these functions and signaling pathways could substantially impact the mitochondrial proteome. Tripartite interactions between the ER-mitochondria–lipid droplets could also play a significant role in forming a functional mitochondrial subpopulation, perhaps specifically in lipid exchange and homeostasis (Najt et al., 2023; Galmes et al., 2016). It would be interesting to perform lipidomic analyses on the isolated mitochondrial subpopulations to determine whether lipid contents differ even if the proteomes are indistinguishable. Another hypothetical organelle that could influence the mitochondrial proteome is the lysosome, as it forms a stable tethered contact with mitochondria and serves as the site of mitochondrial fission (Wong et al., 2018).

A significant advantage of our dual-purification system is the adaptability of spatial resolution using APEX2 labeling systems. In our original strategy, we established six APEX2 fusions of resident membranes on the ER, Golgi, endosome, lysosome, lipid droplet and peroxisome. Other choices to expand this system are the plasma membrane and nuclear membrane, as well as non-membranous organelle structures within the cell like the centrosome, base of the primary cilia, stress granules, and protein aggregates or condensates. This could include APEX2-fusion proteins of OFD1 (base of primary cilium) (Lopes et al., 2011), centrin-2 (centrosome) (Laoukili et al., 2000), Hsp27 (stress granules) (Collier and Schlesinger, 1986; Arrigo et al., 1988) and TIA-1 (stress granules) (Kedersha et al., 1999). It is also plausible that this dual-purification system could be designed to use other proximity labeling measures such as the new iAPEX system to limit hydrogen peroxide damage (Sroka et al., 2025 preprint), TurboID and its derivatives (Branon et al., 2018), BioID (Roux et al., 2012), or a split proximity labeling system, such as split-APEX2 (Han et al., 2019). Of cautionary note, depletion of biotin causes mitochondrial protein hyperacetylation in yeast and heme synthesis and mitochondrial respiration defects in mammalian cells (Madsen et al., 2015; Atamna et al., 2007; Lohr et al., 2020; Ochoa-Ruiz et al., 2015). Still, it might be possible to transiently express these systems to avoid long-term mitochondrial dysfunction.

We envision that this dual purification system will be utilized by other groups to investigate intracellular mitochondrial heterogeneity and that this will be a significant step towards uncovering mitochondrial specialization in a less biased way. Still, there are several limitations to our strategy. Foremost, the 8×His–mCherry– Omp25:Ni-NTA purification is not completely efficient. After six elutions, there remains ∼30–35% of mitochondrial protein still on the Ni-NTA beads (Fig. 1C,D). In the original design of our system, we attempted 4×His, 6×His, 8×His and 10×His purifications. Both 4×His- and 6×His-tagged mitochondria were substantially lost in the wash steps of the protocol. 10×His failed to elute from the beads even upon 1 M imidazole addition. It is possible that increasing the number of elutions from six to eight or ten might yield better elution. Additionally, increasing imidazole concentration from 250 mM to 500 mM might also increase mitochondrial yield. Indeed, replacing (or supplementing) this step with differential centrifugations and sucrose gradients to better purify the mitochondria could prove useful, but would come with the cost of dramatically increased time of purification.

It is also possible that many of the non-mitochondrial contaminants observed in our proteomics data, approximately ∼5000 proteins, are due to non-specific binding to the Ni-NTA and/or streptavidin beads. While imidazole washes likely removed some of these contaminants, it is entirely possible that further optimization or addition of washing steps, including some with high-salt concentrations, might be required for a cleaner purification. As a result, it is difficult to assess which proteins are contaminants versus those proteins that genuinely associate with mitochondria. An additional limitation is the inability to perform functional downstream analyses on the isolated mitochondrial subpopulations. APEX2 proximity labeling requires a concentration of hydrogen peroxide that will likely cause severe mitochondrial damage, even with a relatively short (<1 min) labeling time.

Here, we created a dual-purification system for the investigation of intracellular mitochondrial heterogeneity. Using an elutable mitochondrial outer membrane tag in combination with APEX2 proximity labeling, we captured subpopulations of mitochondria for proteomic analyses. With an adaptable methodology, we foresee our strategy being used to provide significant insights into mitochondrial specialization and its role in cell biology.

## MATERIALS AND METHODS

### Cell culture, retroviral harvest and transduction, stable cell line creation

U-2 OS osteosarcoma and HEK293T cells were obtained from the ATCC. Mycoplasma testing was performed once every other month. Cells were cultured and maintained in Dulbecco's modified Eagle medium (DMEM; Thermo Fisher Scientific, cat. no. MT10013CV) supplemented with 10% FBS (Sigma-Aldrich, cat. no. F0926, batch no. 22M275) in an incubator at 37°C with 5% CO_2_.

To create replication-incompetent retroviruses, HEK293T cells were seeded at 50% density and transfected using polyethylenimine (PEI; Polysciences Inc., cat. no. 23966) at a 4:1 ratio of PEI µl:1 µg of DNA. Cells were transfected with a retroviral transfer plasmid, a packaging vector containing gag-pol, and an envelope plasmid of vsv-g at a 1.5:1:0.5 ratio. 16 h after transfection, medium was exchanged and viruses were propagated for an additional 24 to 48 h. Viruses were harvested, spun down to remove cell debris (1250 ***g*** for 5 min), and filtered through a 0.45 µm filter (VWR International, cat. no. 28145-505). All plasmid details can be found in Table S2.

For transduction, 5% FBS and 10 µg/ml polybrene transfection reagent (Sigma-Aldrich, cat. no. TR-1003-G) was added to retroviral medium prior to addition onto cells to be transduced. After 18 h, viral medium was removed, and cells were allowed to recover for 24 h in non-selection medium before the addition of a selection agent (either 10 µg/ml blasticidin or 1 µg/ml puromycin) for up to 5 days.

For the 8×His–mCherry–Omp25 stable U-2 OS cell line, cells were transduced and selected against blasticidin for 5 days prior to FACS-based sorting to bulk isolate mCherry-positive cells below an mCherry fluorescence threshold set at 10% of the maximum fluorescence. These cells acted as the background population for each APEX2-fusion system.

For the APEX2-fusion systems, 8×His–mCherry–Omp25-positive U-2 OS cells were transduced with one of the APEX2-fusion systems, allowed to recover for 24 h post-transduction, and selected against 1 µg/ml puromycin (Thermo Fisher Scientific, cat. no. A1113803) for up to 5 days prior to FACS-based sorting to bulk isolate the lowest 20% GFP-positive cells (in the background of the mCherry positivity from the above step).

### His-tagged MitoTag-AP and elution

All steps were performed at 4°C. 8×His–mCherry–Omp25 U-2 OS cells were washed with ice-cold PBS, aspirated, and scraped in 1 ml of KPBS (10 mM KH_2_PO_4_, 136 mM KCl, pH 7.25) with phenylmethylsulfonyl fluoride (PMSF) and mammalian protease inhibitor cocktail (mPIC; Sigma-Aldrich, cat. no. P8340). Cells were spun at 1000 ***g*** for 2 min, supernatant aspirated, and the resulting pellet was resuspended in 1 ml of KPBS with PMSF and mPIC. Cell suspension was added to a 2 ml dounce homogenizer and dounced for 35 strokes. Homogenate was collected, spun at 1000 ***g*** for 2 min, and supernatant was collected and added to prewashed 100 µl of HisPur Ni-NTA (Thermo Fisher Scientific, cat. no. 88832) magnetic beads per 3–15 cm plates of cells. Homogenate-bead mixture was incubated with rotation for 10 min, pulse spun at 1000 ***g*** for 5 s, then placed on a magnetic trap for 30 s. Supernatant was removed and magnetic beads were washed with KPBS supplemented with 50 mM imidazole before placing back on magnetic trap. Beads were washed a total of eight times before elution procedure.

Ni-NTA beads (Thermo Fisher Scientific, cat. no. 88832) were then incubated with 100–200 µl of elution buffer (KPBS supplemented with 250 mM imidazole, PMSF, and mPIC) and incubated on a multi-tube vortexer on a low setting. Each elution proceeded for 10 min after which tube was removed from vortexer, beads were collected on a magnetic trap, and supernatant collected. This procedure was repeated a total of six times.

### Immunoblotting

For immunoblotting, 50 µl of 1× Laemmli sample SDS buffer was added, and beads were boiled for 5 min prior to running on SDS-PAGE following standard procedures. Gel was wet transferred onto a 0.45 µm nitrocellulose membrane using a tris-glycine buffer with 20% methanol for 1 h and 40 min at 4°C with ice-pack at 100 V. Membrane was then blocked with either 5% non-fat milk powder in TBST or 5% BSA in TBST for 1 h prior to the addition of denoted antibodies in the respective blocking solution: rabbit anti-TOMM40 (1:2000; Abcam, cat. no. ab185543), mouse anti-SDHA (1:3000; Abcam, cat. no. ab14715), rabbit anti-calreticulin (1:1000; Cell Signaling Technologies, cat. no. 2891) and mouse anti-ATP5A (Abcam, cat. no. ab14748). Donkey anti-mouse-IgG (1:10000; LICOR Bio., cat. no. 928-68072) and donkey anti-rabbit-IgG (1:10000; LICOR Bio., cat. no. 926-32213) secondary antibodies were used. Immunoblots were scanned using a LICOR Odyssey CLx.

### APEX2 labeling and streptavidin pulldown

Within the hour of labeling, a quenching solution consisting of ice-cold 1× PBS (137 mM NaCl, 8 mM Na2HPO4, 2.7 mM KCl, and 1.47 mM KH_2_PO_4_, pH 7.1), 10 mM sodium ascorbate, 10 mM sodium azide, and 5 mM Trolox (Sigma-Aldrich, cat. no. 238813) was created. At 30 min prior to labeling, U-2 OS cells were incubated with the appropriate amount of biotin phenol (Iris Biotech, cat. no. LS-3500.1000). After 30 min, cells were labeled with 1 mM hydrogen peroxide and labeled for the desired time. Medium was quickly aspirated, ice-cold quenching solution was added and left on cells for 1 min. Quenching solution was aspirated and re-applied for a total of three times per plate. Cells were processed as in the ‘His-tagged MitoTag-AP and elution’ protocol with the addition of 10 mM sodium ascorbate, 10 mM sodium azide and 5 mM Trolox in each aforementioned buffer.

After the final elution, 300 µl of prewashed (with KPBS) streptavidin beads were added to the elution and incubated with rotation overnight. Streptavidin beads were pulse spun and placed on a magnetic trap, and supernatant was removed. Beads were washed for a total of four times.

For immunoblotting, 50 µl of 1× Laemmli sample SDS buffer was added, and beads were boiled for 5 min prior to running on SDS-PAGE. For proteomics, 50 µl of 200 mM HEPPS, 8 M urea, 1% SDS, and 1% Triton X-100 lysis buffer was added to the beads, incubated for 20 min at room temperature, supernatant collected, and the step was repeated to collect a total of 100 µl.

### Live-cell imaging and immunofluorescence

Live-cells were imaged with a Zeiss LSM 880 confocal microscope with Airyscan with 37°C incubation and 5% CO_2_. Green fluorescent tagged proteins were captured on a 488 nm channel and mCherry tagged proteins were captured on a 561 nm channel. For live-cell MitoTracker Green (cat. no.: M46750; Invitrogen), cells were incubated with 50 nM MitoTracker Green for 30 min, washed with 1× PBS and medium replaced.

For immunofluorescence, cells were labeled with the designated biotin phenol concentration and labeled with hydrogen peroxide for the denoted time. Cells were quenched three times with quenching solution (see ‘APEX2 labeling and streptavidin pulldown’ section). Cells were then fixed with 4% paraformaldehyde in 1× PBS for 15 min and washed three times with PBS prior to permeabilizing with ice-cold 100% methanol for 5 min at −20°C. Fixed samples were blocked for 2 h with 3% bovine serum albumin (BSA), fraction V, fatty acid free (Millipore Sigma, cat. no. 10775835001) at 4°C. Immunostaining was performed with streptavidin conjugated to Alexa Fluor 647 (Thermo Fisher Scientific, cat. no. S32357) for 1 h at room temperature, washed four times with 1× PBS and staged with ProLong Gold with DAPI. Images were captured on a Zeiss LSM 880.

### Mitochondrial proteinase K assay

Following imidazole elution, eluted mitochondria were equally distributed to six tubes and spun at 10,000 ***g*** for 5 min to pellet. Supernatant was aspirated and resulting mitochondrial pellets were resuspended in one of the following buffers: isotonic (225 mM mannitol, 75 mM sucrose, 1 mM EGTA pH 7.4, 10 mM HEPES pH 7.4), hypotonic (10 mM HEPES, pH 7.4), or detergent (isotonic with 1% Triton X-100), all with or without 20 µg/ml Proteinase K (New England Biolabs, cat. no. P8107S). Cells were incubated on ice for 5 min before adding PMSF to a final concentration of 5 mM to all samples. Cells were incubated on ice for an additional 5 min before isotonic and hypotonic (with or without Proteinase K) samples were spun at 10,000 ***g*** for 5 min. After centrifugation, supernatant was carefully aspirated, pellets were resuspended in their respective buffers with PMSF and without Proteinase K. All samples were then boiled for 5 min prior to running on SDS-PAGE gels following standard procedures. Antibodies used were: Tomm20 (1:1000; Cell Signaling Technologies, cat. no. 42406), Smac (also known as Diablo) (1:1000; Cell Signaling Technologies, cat. no. 2954), HtrA2/Omi (1:1000; Cell Signaling Technologies, cat. no. 9745) and Hsp60 (1:2000; Cell Signaling Technologies, cat. no. 12165). Secondary antibodies were used as previously mentioned.

### Sample preparation for mass spectrometry

Samples for protein analysis were prepared essentially as previously described (Navarrete-Perea et al., 2018). 5 µg of each proteome was reduced with 5 mM TCEP. Cysteine residues were alkylated using 10 mM iodoacetimide for 20 min at room temperature (RT) in the dark. Excess iodoacetimide was quenched with 10 mM DTT. A buffer exchange was carried out using a modified SP3 protocol (Hughes et al., 2019). Briefly, ∼250 µg of Cytiva SpeedBead Magnetic Carboxylate Modified Particles (65152105050250 and 4515210505250), mixed at a 1:1 ratio, were added to each sample. 100% ethanol was added to each sample to achieve a final ethanol concentration of at least 50%. Samples were incubated with gentle shaking for 15 min. Samples were washed three times with 80% ethanol. Protein was eluted from SP3 beads using 200 mM HEPPS pH 8.5 containing Lys-C (Wako, 129-02541). Samples were digested overnight at room temperature with vigorous shaking. The next morning trypsin was added to each sample and further incubated for 6 h at 37° C. Acetonitrile was added to each sample to achieve a final concentration of ∼33%. Each sample was labelled, in the presence of SP3 beads, with ∼62.5 µg of TMTPro reagents (Thermo Fisher Scientific). Following confirmation of satisfactory labelling (>97%), excess TMT was quenched by addition of hydroxylamine to a final concentration of 0.3%. The full volume from each sample was pooled and acetonitrile was removed by vacuum centrifugation for 1 h. The pooled sample was acidified, and peptides were de-salted using a Sep-Pak 50 mg tC18 cartridge (Waters). Peptides were eluted in 70% acetonitrile, 1% formic acid and dried by vacuum centrifugation and fractionated using a Pierce High pH Reversed-Phase Peptide Fractionation kit (Thermo Fisher Scientific). A total of eight fractions were collected for LC-MS/MS analysis.

### Liquid chromatography separation and tandem mass spectrometry

Proteome data were collected on an Orbitrap Fusion Lumos mass spectrometer (Thermo Fisher Scientific) coupled to a Proxeon EASY-nLC 1000 LC pump (Thermo Fisher Scientific). Fractionated peptides were separated using a 180 min gradient at 500 nl/min on a 35 cm column (i.d. 100 μm, Accucore, 2.6 μm, 150 Å) packed in-house. MS1 data were collected in the Orbitrap (120,000 resolution; maximum injection time 50 ms; AGC 10×10^5^). Charge states between 2 and 5 were required for MS2 analysis, and a 180 s dynamic exclusion window was used. Top 10 MS2 scans were performed in the ion trap with CID fragmentation (isolation window 0.5 Da; Rapid; NCE 35%; maximum injection time 35 ms; AGC 1.2×10^4^). An on-line real-time search algorithm (Orbiter) was used to trigger MS3 scans for quantification (Schweppe et al., 2020). MS3 scans were collected in the Orbitrap using a resolution of 50,000, NCE of 55%, maximum injection time of 200 ms, and AGC of 3.0×10^5^. The close out was set at two peptides per protein per fraction (Schweppe et al., 2020).

### Data analysis

Raw files were converted into mzXML format, and monoisotopic peaks were re-assigned using Monocle (Rad et al., 2021). Searches were performed using the Comet search algorithm against a human database downloaded from Uniprot in February 2020. We used a 50 ppm precursor ion tolerance, 1.0005 fragment ion tolerance and 0.4 fragment bin offset for MS2 scans collected in the ion trap. TMTpro on lysine residues and peptide N-termini (+304.2071 Da) and carbamidomethylation of cysteine residues (+57.0215 Da) were set as static modifications, whereas oxidation of methionine residues (+15.9949 Da) was set as a variable modification.

Each run was filtered separately to 1% false discovery rate (FDR) on the peptide-spectrum match (PSM) level. Then proteins were filtered to the target 1% FDR level across the entire combined data set. For reporter ion quantification, a 0.003 Da window around the theoretical *m/z* of each reporter ion was scanned, and the most intense *m/z* was used. Reporter ion intensities were adjusted to correct for isotopic impurities of the different TMTpro reagents according to manufacturer specifications. Peptides were filtered to include only those with a summed signal-to-noise (S/N)≥160 across all TMT channels. The signal-to-noise (S/N) measurements of peptides assigned to each protein were summed (for a given protein). These values were normalized so that the sum of the signal for all proteins in each channel was equivalent thereby accounting for equal protein loading. For volcano plots, TMT protein quantifications were log transformed, and analyzed by group comparison multiple *t*-tests with two-stage step up method of Benjamini, Krieger and Yekutieli on Graphpad Prism 10 (Table S1).

## Supplementary Material



10.1242/joces.263693_sup1 Supplementary information

Table S1.
