# Supplementary material for: A dual-purification system to isolate mitochondrial subpopulations
Source: J Cell Sci. 2025 Apr 14;138(9):jcs263693. doi: 10.1242/jcs.263693 (PMC12045638; doi:10.1242/jcs.263693)
Supplement: Supplementary information [file joces-138-263693-s1.pdf]

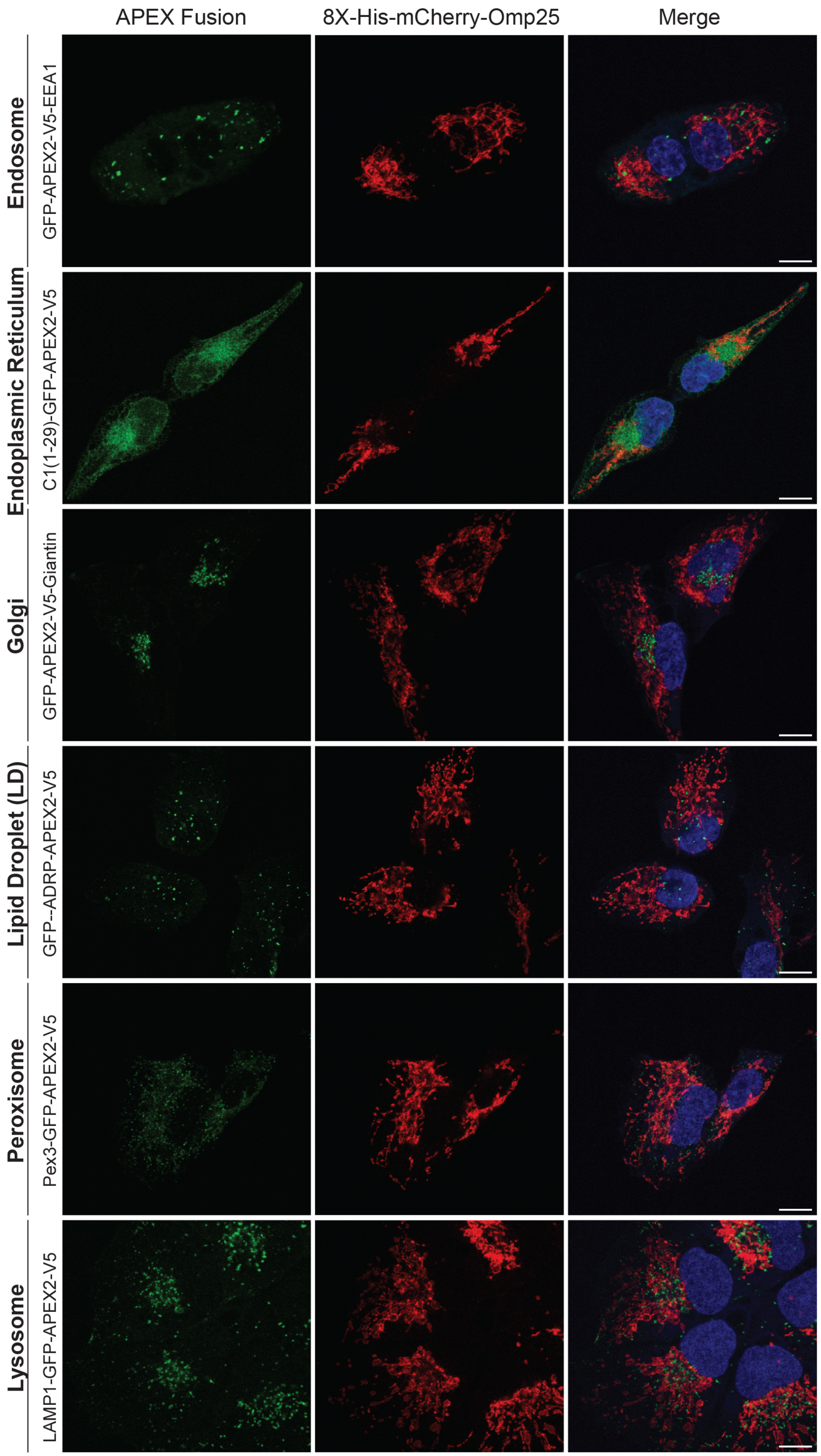


**Fig. S1. Live-cell imaging of dual-purification systems.** Live-cell imaging of U-2 OS cells stably expressing 8x-His-mCherry-Omp25 and the indicated APEX2-fusion protein on the listed organelle.

Journal of Cell Science • Supplementary information

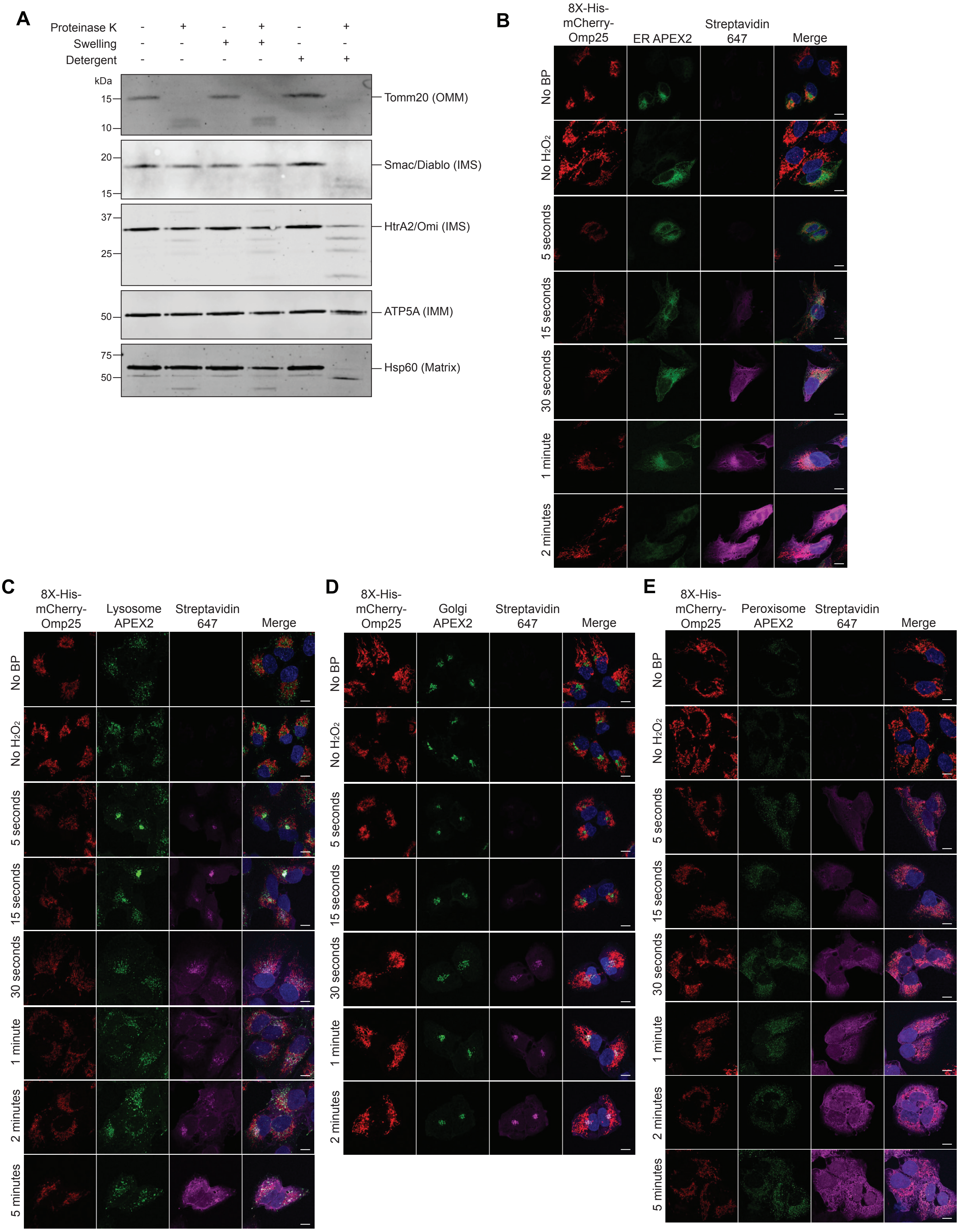


**Fig. S2. Time trials of APEX2-fusion labeling specificity.** *A*, mitochondrial proteinase K protection and swelling assay. Eluted mitochondria were subjected to proteinase K alone, proteinase K with hypotonic buffer, or proteinase K with detergent (n=5 independent trials). *B*, as in Figure 2G, but with the Endoplasmic Reticulum (C1(1-29))-APEX2 system and with 200 µM biotin phenol for the indicated time points. *C*, as in *B*, but with the Lysosome (LAMP1)-APEX2 system and 5 µM biotin phenol for the indicated time points. *D*, as in *B*, but with the Golgi (Giantin)-APEX2 system and 25 µM biotin phenol for the indicated time points. *E*, as in *B*, but with the Peroxisome (Pex3)-APEX2 system and 10 µM biotin phenol for the indicated time points.

Journal of Cell Science • Supplementary information

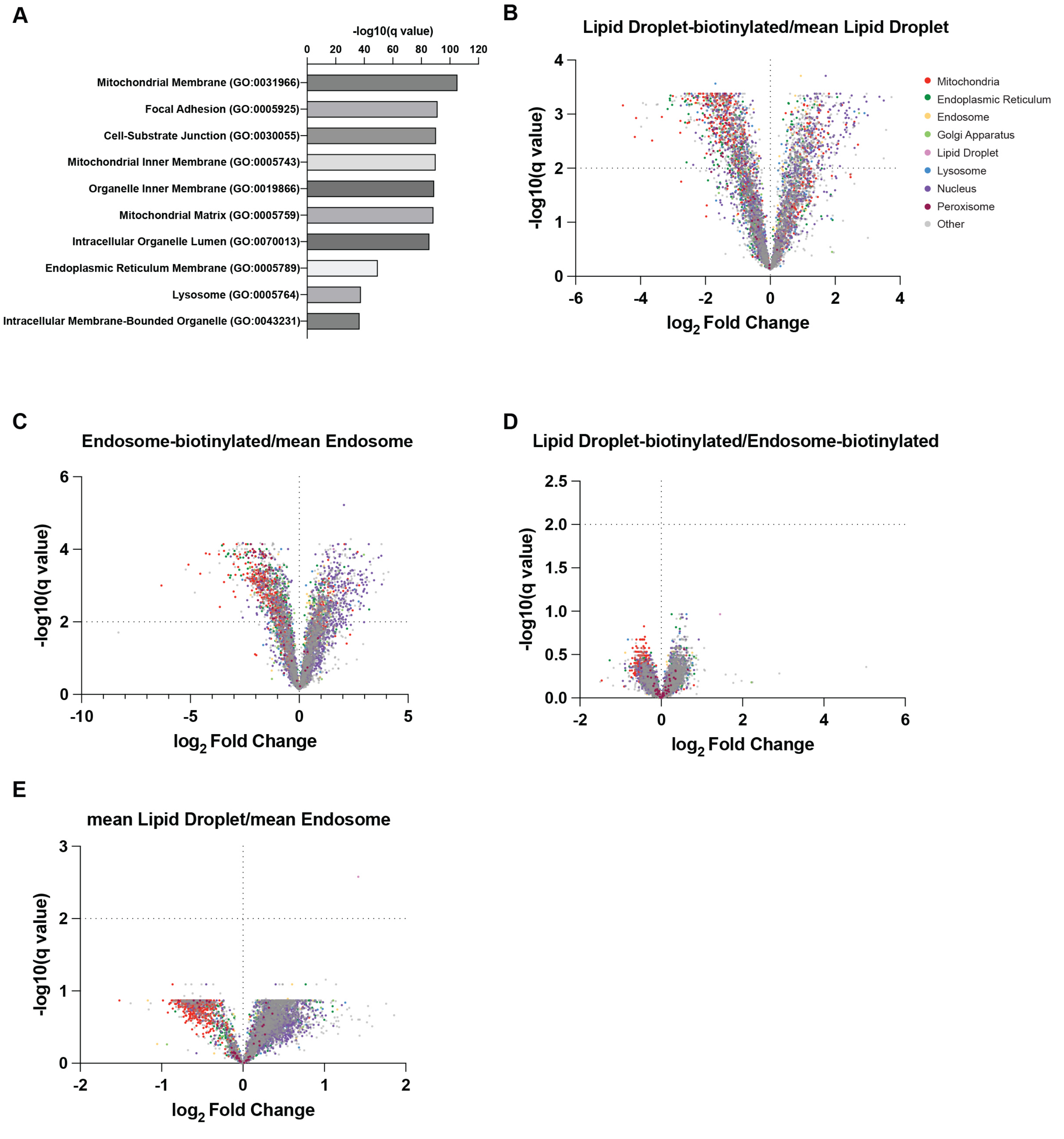


Journal of Cell Science • Supplementary information

**Fig. S3. Volcano plots of all detected proteins resolved in the TMT quantitative proteomics dataset.** *A,* Gene Ontology Cellular Component analysis of total proteomics dataset. Top ten GOCC term q-values plotted. *B*, volcano plot of Figure 3C of all proteins detected by TMT quantitative proteomics. Ten de-enriched proteins labeled in blue and ten enriched proteins labeled in red. *C*, volcano plot of Figure 3D of all proteins detected by TMT quantitative proteomics. Ten de-enriched proteins labeled in blue and ten enriched proteins labeled in red. *D*, volcano plot of Figure 3E of all proteins detected by TMT quantitative proteomics. Top labeled proteins are not-significant. *E*, volcano plot of Figure 3F of all proteins detected by TMT quantitative proteomics. With the exception of PLIN2, top labeled proteins are not-significant. Data presented in volcano plots were FDR corrected using two-stage step-up method of Benjamini, Krieger, and Yekutieli method.

**Table S1.**

Available for download at
https://journals.biologists.com/jcs/article-lookup/doi/10.1242/jcs.263693#supplementary-data

Journal of Cell Science • Supplementary information

## Table S2. Plasmids used within this study

| Plasmids | Source | Citation |
|---|---|---|
| pMXs-3XHA-EGFP-OMP25 | Addgene #83356 | (Chen et al., 2016) |
| pMXs-8XHis-mCherry-OMP25 | This study | N/A |
| Gag/pol | Addgene #14887 | (Reya et al., 2003) |
| pCMV-VSV-G | Addgene #8454 | (Stewart et al., 2003) |
| C1(1-29)-TurboID-V5-pCDNA3 | Addgene #107173 | (Branon et al., 2018) |
| pERB264 (Pex3-GFP-Halo) | Addgene #67764 | (Ballister et al., 2015) |
| LAMP1-mGFP | Addgene #34831 | (Falcon-Perez et al., 2005) |
| GFP-EEA1-wt | Addgene #42307 | (Lawe et al., 2000) |
| pmScarlet-H_Giantin_C1 | Addgene #85049 | (Bindels et al., 2017) |
| pEGFP-C1-ADRP | Addgene #87161 | (Salo et al., 2016) |
| ERM-APEX2 | Addgene #79055 | (Lam et al., 2015) |
| pQCXIP-C1(1-29)-EGFP-APEX2-V5 | This study | N/A |
| pQCXIP-Pex3-EGFP-APEX2-V5 | This study | N/A |
| pQCXIP-LAMP1-EGFP-APEX2-V5 | This study | N/A |
| pQCXIP-EGFP-APEX2-V5-EEA1 | This study | N/A |
| pQCXIP-EGFP-APEX2-V5-Giantin | This study | N/A |
| pQCXIP-EGFP-ADRP-APEX2-V5 | This study | N/A |

Ballister, E. R., Ayloo, S., Chenoweth, D. M., Lampson, M. A. and Holzbaur, E. L. F. (2015). Optogenetic control of organelle transport using a photocaged chemical inducer of dimerization. *Curr. Biol.* 25, R407-R408. doi:10.1016/j.cub.2015.03.056

Bindels, D. S., Haarbosch, L., van Weeren, L., Postma, M., Wiese, K. E., Mastop, M., Aumonier, S., Gotthard, G., Royant, A., Hink, M. A. et al. (2017). mScarlet: a bright monomeric red fluorescent protein for cellular imaging. *Nat. Methods* 14, 53-56. doi:10.1038/nmeth.4074

Branon, T. C., Bosch, J. A., Sanchez, A. D., Udeshi, N. D., Svinkina, T., Carr, S. A., Feldman, J. L., Perrimon, N. and Ting, A. Y. (2018). Efficient proximity labeling in living cells and organisms with TurboID. *Nat. Biotechnol.* 36, 880-887. doi:10.1038/nbt.4201

Journal of Cell Science • Supplementary information

Chen, W. W., Freinkman, E., Wang, T., Birsoy, K. and Sabatini, D. M. (2016). Absolute quantification of matrix metabolites reveals the dynamics of mitochondrial metabolism. *Cell* 166, 1324-1337.e11. doi:10.1016/j.cell.2016.07.040

Falcon-Perez, J. M., Nazarian, R., Sabatti, C. and Dell'Angelica, E. C. (2005). Distribution and dynamics of Lamp1-containing endocytic organelles in fibroblasts deficient in BLOC-3. *J. Cell Sci.* 118, 5243-5255. doi:10.1242/jcs.02633

Lam, S. S., Martell, J. D., Kamer, K. J., Deerinck, T. J., Ellisman, M. H., Mootha, V. K. and Ting, A. Y. (2015). Directed evolution of APEX2 for electron microscopy and proximity labeling. *Nat. Methods* 12, 51-54. doi:10.1038/nmeth.3179

Lawe, D. C., Patki, V., Heller-Harrison, R., Lambright, D. and Corvera, S. (2000). The FYVE domain of early endosome antigen 1 is required for both phosphatidylinositol 3-phosphate and Rab5 binding. Critical role of this dual interaction for endosomal localization. *J. Biol. Chem.* 275, 3699-3705. doi:10.1074/jbc.275.5.3699

Reya, T., Duncan, A. W., Ailles, L., Domen, J., Scherer, D. C., Willert, K., Hintz, L., Nusse, R. and Weissman, I. L. (2003). A role for Wnt signalling in self-renewal of haematopoietic stem cells. *Nature* 423, 409-414. doi:10.1038/nature01593

Salo, V. T., Belevich, I., Li, S., Karhinen, L., Vihinen, H., Vigouroux, C., Magré, J., Thiele, C., Hölttä-Vuori, M., Jokitalo, E. et al. (2016). Seipin regulates ER-lipid droplet contacts and cargo delivery. *EMBO J.* 35, 2699-2716. doi:10.15252/embj.201695170

Stewart, S. A., Dykxhoorn, D. M., Palliser, D., Mizuno, H., Yu, E. Y., An, D. S., Sabatini, D. M., Chen, I. S., Hahn, W. C., Sharp, P. A. et al. (2003). Lentivirus-delivered stable gene silencing by RNAi in primary cells. *RNA* 9, 493-501. doi:10.1261/rna.2192803

Journal of Cell Science • Supplementary information
