# Supplementary material for: A dual-purification system to isolate mitochondrial subpopulations
Source: J Cell Sci. 2025 Apr 14;138(9):jcs263693. doi: 10.1242/jcs.263693 (PMC12045638; doi:10.1242/jcs.263693)
Supplement: Supplementary file 1 [file joces-138-263693_review_history.pdf]

Journal of Cell Science

A dual-purification system to isolate mitochondrial subpopulations

Corey N. Cunningham, Jonathan G. Van Vranken, Jakeline Larios, Katarina Heyden, Steven P. Gygi, Jared Rutter

DOI: 10.1242/jcs.263693

Editor: Heidi McBride

Review timeline

Original submission:	4 November 2024
Editorial decision:	25 November 2024
First revision received:	30 January 2025
Accepted:	26 February 2025

Original submission

<u>First decision letter</u>

MS ID#: jcs.263693

MS TITLE: A dual-purification system to isolate mitochondrial subpopulations
AUTHORS: Corey N. Cunningham; Jonathan G. Van Vranken; Jakeline Larios; Katarina Heyden; Steven P. Gygi; Jared Rutter

We have now reached a decision on the above manuscript.

To see the reviewers' reports and a copy of this decision letter, please go to:

As you will see, the reviewers gave favourable reports but raised some points that will require amendments to your manuscript. I hope that you will be able to carry these out because I would like to be able to accept your paper, depending on further comments from reviewers.

Please ensure that you clearly highlight all changes made in the revised manuscript. Please avoid using 'Tracked changes' in Word files as these are lost in PDF conversion.

I should be grateful if you would also provide a point-by-point response detailing how you have dealt with the points raised by the reviewers in the 'Response to Reviewers' box. Please attend to all of the reviewers' comments. If you do not agree with any of their criticisms or suggestions please explain clearly why this is so.

<u>Reviewer 1</u>

Advance summary and potential significance to field

Cunningham et al describe a novel method to isolate pools of mitochondria associated with different organelles from the same cell line. To do this they generate constructs targeting a cytosol facing APEX2 to various organelle surfaces in the background of cells expressing a variant of MitoTag (HIS fused to Omp25). The idea is to add the substrate biotin-phenol thereby biotinylating proteins on the outer membrane of associated mitochondria, purify crude mitochondria using the HIS tag, then purify the biotinylated mitochondria via streptavidin. They generate constructs and test localisation of a large number of APEX2 constructs and perform the double purification on two targeting the endosome and lipid droplets respectively. The proteomes of the resulting isolates are

© 2025. Published by The Company of Biologists under the terms of the Creative Commons Attribution License (https://creativecommons.org/licenses/by/4.0/).

examined. Overall this is study of interest to mito biology researchers and I will consider trying something like this in my own group. My criticism is that there is minimal analysis of the purified organelles, both in respect to their integrity and proteomic differences, the latter being fairly superficial and mostly qualitative in nature.

Comments for the author

Major comments
- How does the use of ATP5A in the Fig. 2A-F western blotting experiments confirm that mitochondria are intact? What about IMS proteins? It would be more appropriate to to a protease protection (+/- swelling) assay on the eluates. One wouldn't need to do this with every construct, just a representative experiment would suffice.

- The analysis of the proteomics data is fairly limited and in my opinion a bit of a missed opportunity. Moreover the relative levels of enrichment are only considered in qualitative way (eg on P2 line 43-37 the authors highlight how some of the mito. protein synthesis apparatus is more enriched in EEA1 associated mitos). Instead of labelling cherry picked proteins on volcano plots, it would make sense to do enrichment analysis (best option would be the Mitopathways terms in Mitocarta3.0). It would also be possible to use GOCC on the non-filtered data in Fig. S3 to discuss the nature of non-mito proteins co-isolating with purified mitochondria. Although still qualitative, even changing the color of the dots based on GOCC annotation would be informative.

- Related to the last point, as authors only consider the level of enrichment against the mean mitochondria there is never a 'definitive' list of proteins for each isolation. Without this how can one be sure that a protein is a contaminant (eg by binding to the Ni-NTA column)? The best way to do this would be to have a third comparison of cells not expressing Mitotag/HIS-Omp25. It might be impractical to do this for a revision, but I would urge discussion of this caveat at a minimum.

-Finally, the authors should include supplementary data tables (eg xls files) for the proteomics data underpinning the figures (ie the protein names against reporter intensities for the whole dataset, plus results of the tests used in generating the volcano plots) and upload the .raw data to a repository like PRIDE. As this is presented as a new method it would be also nice if the plasmids were made available to the community via Addgene.

Minor comments
-It was never clear to me why the authors switched the MitoTag system from HA to HIS? It is possible to elute from HA using HA peptide, which I imagine is less problematic for downstream analysis compared to imidazole. Does the 50mM imidazole used have a predicted impact on either mito integrity or function that may impact the results?

-Methods: origin of plasmids and retroviral system (eg addgene codes, citation) should be included. P13 line 9 suggests there are two APEX2 fusion systems. What does this mean?

-Fig. 1A is not super intuitive and I can make a few suggestions:1) on the schematic of a cell it would be helpful to indicate which organelles were tagged with the APEX2 construct (rather than relying on the inset table alone)3; 2) a symbol like '+' could be used to indicate the addition of biotin-phenol in the inset labelled (1); 3) unobvious visual elements should be labelled eg. the 50nm cloud of activated.

-The organelle targeted by the APEX2 constructs should be indicated on the figures themselves (rather than rely on the reader knowing the localisation of the tagged protein) throughout the manuscript. Alternately this could be included in the legend, but in my view on the figure is more intuitive.

-Fig S1 & S2 these lacks a scale bars. This brightness of the imaging here could be improved and made to be comparable with the main figure imaging results.

-Fig S3: for ease of reader interpretation the populations of Mitocart3.0 and other proteins should be indicated on the figure, eg through colored dots or empty circles vs black dots.

© 2025. Published by The Company of Biologists under the terms of the Creative Commons Attribution License (https://creativecommons.org/licenses/by/4.0/).

Reviewer 2

Advance summary and potential significance to field

This manuscript by Cunningham et al describes an approach to the isolation of mitochondrial subpopulations on the basis of their interactions with other organelles. The underlying assumption of the work is that mitochondria that participate in membrane contacts sites with e.g. lipid droplets or endosomes may have specialized functions and, as a result, distinctive proteomes. The authors deploy an APEX2 biotinylation and avidin elution strategy in cells in which mitochondria were labeled with MitoTag to enrich for subpopulations. They succeeded in isolating mitochondrial subpopulations associated with LDs and endosomes, respectively, which exhibited proteomes distinct from the total mito pool, though not significantly different from each other. Overall this is a useful contribution to the literature, particularly the nuances of tuning the APEX labeling and elution protocols to achieve optimal subpopulation enrichment. I have some minor comments, described below.

Comments for the author

- It's not clear from the abstract that the proteomes of the LD and endosome-associated mitos are indistinguishable (by these methods and statistics, in these cells). The abstract needs editing to better reflect the results.
- pg 4 line 10. This paragraph would benefit from the addition of PMID: 39506109. This recent work demonstrates that mito subpopulations can be both compositionally and metabolically distinct.
- pg 7 line 18 re: hydrogen peroxide "data not shown"; this is not acceptable. All data mentioned in the manuscript must be available to the reader.
- The formation and functions of tripartite membrane contacts between mito, ER, and LD are missing from the Discussion. The manuscript would benefit from the addition of a few sentences describing such, and their potential relevance to the Results. See PMCID: PMC5278607 and PMCID: PMC10278152, reviewed in PMID: 38944117.
- A minor quibble: the cartoon of organelles presented in Fig 1A is inaccurate regarding mito and ER morphology, as well as the presence and abundance of the contact sites between the respective organelles. Please see e.g. PMID: 38242082.
- The 8x-His-mCherry-OMP25 and Mitotracker in Fig 1B are not as colocalized as I had expected. What is the rationale for the presence of MTGreen puncta without mCherry signal?
- The images in Supplemental Fig2 are too small to discern clearly. Fig 2G and 2H are borderline.

First revision

Author response to reviewers' comments

Response to Reviewers

Reviewer #1:

1. How does the use of ATP5A in the Fig. 2A-F western blotting experiments confirm that mitochondria are intact? What about IMS proteins? It would be more appropriate to a protease protection (+/- swelling) assay on the eluates. One wouldn't need to do this with every construct, just a representative experiment would suffice.

   -*We thank the reviewer for this suggestion. We have now performed this experiment (n=5) and included it as Supplemental Figure 2A. As shown, proteinase K alone acts primarily on outer mitochondrial membrane protein Tomm20 (OMM) with minor cleavage of HtrA2/Omi (IMS), indicating that a small subset of mitochondria have a disruption of the OMM. With hypotonicity (swelling), Smac/Diablo (IMS) and HtrA2/Omi (IMS) show decreases and some cleavage. Upon detergent and proteinase K treatment, all proteins are accessible for cleavage. Together, these data suggest that the majority of the eluted mitochondria are*

© 2025. Published by The Company of Biologists under the terms of the Creative Commons Attribution License (https://creativecommons.org/licenses/by/4.0/).

mostly intact with a percentage having a slightly disrupted outer membrane. We have also modified the text to reflect these new findings.

2. The analysis of the proteomics data is fairly limited and in my opinion a bit of a missed opportunity. Moreover the relative levels of enrichment are only considered in qualitative way (eg on P2 line 43-37 the authors highlight how some of the mito. protein synthesis apparatus is more enriched in EEA1 associated mitos). Instead of labelling cherry picked proteins on volcano plots, it would make sense to do enrichment analysis (best option would be the Mitopathways terms in Mitocarta3.0). It would also be possible to use GOCC on the non-filtered data in Fig. S3 to discuss the nature of non-mito proteins co-isolating with purified mitochondria. Although still qualitative, even changing the color of the dots based on GOCC annotation would be informative.

   -*We agree that our original analysis was designed to highlight interesting proteins rather than provide a comprehensive picture. We have now adjusted the volcano plots to highlight MitoPathway terms in Mitocarta 3.0, according to their hierarchy (see Figure 3 volcano plots and the GOCC annotation in Supplemental Figure 3 volcano plots).*

3. Related to the last point, as authors only consider the level of enrichment against the mean mitochondria there is never a 'definitive' list of proteins for each isolation. Without this how can one be sure that a protein is a contaminant (eg by binding to the Ni-NTA column)? The best way to do this would be to have a third comparison of cells not expressing Mitotag/HIS-Omp25. It might be impractical to do this for a revision, but I would urge discussion of this caveat at a minimum.

   -*We agree that this is an important point but unfortunately we are unable to perform this additional proteomics experiment. Still, as suggested, we now include a discussion of these points on page 11. Briefly, we agree that it is possible that many of the contaminants we observe in our proteomics are due to non-specific binding to either the Ni-NTA beads or immobilized avidin. Our protocol may require further optimization of washing conditions.*

4. Finally, the authors should include supplementary data tables (eg xls files) for the proteomics data underpinning the figures (ie the protein names against reporter intensities for the whole dataset, plus results of the tests used in generating the volcano plots) and upload the .raw data to a repository like PRIDE. As this is presented as a new method it would be also nice if the plasmids were made available to the community via Addgene.

   - *We have now included the TMT quantitative proteomics dataset as a supplemental table S1, as well as the results of the tests used in generating the volcano plots. As we originally planned, we are in the process of depositing the plasmids to Addgene for community use.*

5. -It was never clear to me why the authors switched the MitoTag system from HA to HIS? It is possible to elute from HA using HA peptide, which I imagine is less problematic for downstream analysis compared to imidazole. Does the 50mM imidazole used have a predicted impact on either mito integrity or function that may impact the results?

   -*We originally built our system using the 3X HA MitoTag system but we were not successful in elution steps using a 1X and even a 3X HA peptide. Also, in terms of costs and elutability, we believe the 8X His MitoTag system is dramatically superior. HA being a peptide also has the potential of creating issues for downstream proteomics. On the other hand, the imidazole elutions did not cause major issues for the proteomics, but may cause a slight disruption of the outer mitochondrial membrane. We expect the APEX2 to cause problems with mitochondrial function as it uses hydrogen peroxide (we have not tested whether imidazole causes similar issues). In our discussion, we do list the caveats of other biotinylation systems, which if optimized correctly, could be substituted for the APEX2.*

6. Methods: origin of plasmids and retroviral system (eg addgene codes, citation) should be included. P13 line 9 suggests there are two APEX2 fusion systems. What does this mean?

© 2025. Published by The Company of Biologists under the terms of the Creative Commons Attribution License (https://creativecommons.org/licenses/by/4.0/).

-*We have now included the origin of all the plasmids and retroviral systems in our materials and methods, and their proper citations (see Table 1). We apologize for the confusing language and have now fixed that statement to properly reflect that there is only one APEX2 fusion system per cell.*

7. Fig. 1A is not super intuitive and I can make a few suggestions:1) on the schematic of a cell it would be helpful to indicate which organelles were tagged with the APEX2 construct (rather than relying on the inset table alone)3; 2) a symbol like '+' could be used to indicate the addition of biotin-phenol in the inset labelled (1); 3) unobvious visual elements should be labelled eg. the 50nm cloud of activated.

   -*We thank the reviewer for these helpful suggestions. 1) We have now labeled the organelles within the cell schematic on the left and include only those for which we've designed the dual-purification system; 2) Since we test all of the APEX2 constructs in Figure 2 and Supplemental Figure 2, we did not include this (+) sign; 3) We have labeled all the non-obvious visual elements in the figure such as the biotin cloud.*

8. The organelle targeted by the APEX2 constructs should be indicated on the figures themselves (rather than rely on the reader knowing the localisation of the tagged protein) throughout the manuscript. Alternately this could be included in the legend, but in my view on the figure is more intuitive.

   -*We agree that the protein-APEX2 fusion is a confusing and now have switched the figure language to highlight the organelle which is being targeted with the APEX2 fusion.*

9. Fig S1 & S2 these lacks a scale bars. This brightness of the imaging here could be improved and made to be comparable with the main figure imaging results.

   -*We have added the scale bars to all immunofluorescence images and also increased the brightness of the supplemental figures to comparable levels with the main figures.*

10. Fig S3: for ease of reader interpretation the populations of Mitocart3.0 and other proteins should be indicated on the figure, eg through colored dots or empty circles vs black dots.

    -*We have highlighted the mitochondrial populations in Figure S3 using GOCC terminology.*

Reviewer #2

11. -It's not clear from the abstract that the proteomes of the LD and endosome-associated mitos are indistinguishable (by these methods and statistics, in these cells). The abstract needs editing to better reflect the results.

    -*We have edited the abstract to better reflect the results.*

12. pg 4 line 10. This paragraph would benefit from the addition of PMID: 39506109. This recent work demonstrates that mito subpopulations can be both compositionally and metabolically distinct.

    -*This reference was published just after our original submission. We are excited to include discussion of this article in our manuscript (see page 3).*

13. pg 7 line 18 re: hydrogen peroxide "data not shown"; this is not acceptable. All data mentioned in the manuscript must be available to the reader.

    -*We apologize for not including this data in our original submission, as we found this data repetitive with the (-) biotin phenol data. We have now included these data in Figure 2 and supplemental figure 2.*

© 2025. Published by The Company of Biologists under the terms of the Creative Commons Attribution License (https://creativecommons.org/licenses/by/4.0/).

14. The formation and functions of tripartite membrane contacts between mito, ER, and LD are missing from the Discussion. The manuscript would benefit from the addition of a few sentences describing such, and their potential relevance to the Results. See PMCID: PMC5278607 and PMCID: PMC10278152, reviewed in PMID: 38944117.

    -*We agree that discussion of the tripartite membrane contacts is valuable and we have now included these references in the discussion.*

15. A minor quibble: the cartoon of organelles presented in Fig 1A is inaccurate regarding mito and ER morphology, as well as the presence and abundance of the contact sites between the respective organelles. Please see e.g. PMID: 38242082.

    -*We appreciate this comment and fully agree that ER morphology is not what is typically represented in Biorender. We've now attempted to make our cell illustration more accurate in regards to ER morphology, inspired by PMID 3824082. We have now labeled the organelles for better identification by the reader and also included more organelle contacts to better reflect the literature.*

16. The 8x-His-mCherry-OMP25 and Mitotracker in Fig 1B are not as colocalized as I had expected. What is the rationale for the presence of MTGreen puncta without mCherry signal?

    -*Upon revisiting our data, we discovered that the image we used was not representative of the images in our dataset. There is still mCherry signal where those MTGreen puncta are located but the signal is weaker. Still, to avoid any confusion for our audience, we have included a more representative image, and one of higher resolution, of the mCherry and MTGreen localization.*

17. The images in Supplemental Fig2 are too small to discern clearly. Fig 2G and 2H are borderline.

    -*We have increased the size of Fig S2 to better match Figure 2H and 2I (revised 2G and 2H). Unfortunately, due to the extent of the time courses, we are unable to make these figures any larger.*

Second decision letter

MS ID#: jcs.263693

MS TITLE: A dual-purification system to isolate mitochondrial subpopulations

AUTHORS: Corey N. Cunningham; Jonathan G. Van Vranken; Jakeline Larios; Katarina Heyden; Steven P. Gygi; Jared Rutter

I am happy to tell you that your manuscript has been accepted for publication in Journal of Cell Science, pending standard publication integrity checks.

Reviewer 1

Advance summary and potential significance to field

The authors have addressed my concerns. The revised manuscript represents a valuable contribution to the field and I look forward to seeing they tools they have developed used in other studies.

© 2025. Published by The Company of Biologists under the terms of the Creative Commons Attribution License (https://creativecommons.org/licenses/by/4.0/).

Reviewer 2

Advance summary and potential significance to field

Overall the authors have addressed my concerns. I have a one very minor suggestion for the text, and one question about material availability:

Lines 39-53 - thank you for the addition of the reference to Ryu et al 2024, however as written the new text interrupts the flow of the main text. This section could benefit from the addition of an explicit statement that the approach described in the paper would be useful to characterize the P45CS-positive mito subpopulation.

Will the APEX2 plasmids generated for this study listed in Table 1 be made available via Addgene?

© 2025. Published by The Company of Biologists under the terms of the Creative Commons Attribution License (https://creativecommons.org/licenses/by/4.0/).
